# Survey on the management of patients treated with therapeutic hypothermia post cardiac arrest in London hospitals

**DOI:** 10.1186/cc10891

**Published:** 2012-03-20

**Authors:** A Walecka, SC Robert, A Prasad

**Affiliations:** 1 The Royal Free Hospital, London, UK

## Introduction

The second UK national survey on therapeutic hypothermia (TH) post cardiac arrest demonstrated an impressive increase in its implementation across the UK (from 28% to 85.6%) [1]. Therapeutic hypothermia, however, induces numerous physiological and pathophysiological changes and therefore should be performed in a standardised and controlled manner in order to be safe and effective. We carried out a telephone survey to determine the current TH practice in London hospitals.

## Methods

Thirty-two London intensive care units (ITUs) were contacted by telephone. The data were analysed using Excel spreadsheets.

## Results

Of the 32 ITUs contacted, 30 (93.7%) had been using therapeutic hypothermia following cardiac arrest. Fifteen (50%) of them were teaching hospitals and the remaining 15 (50%) were district general hospitals. Twenty-two (73.3%) hospitals had a protocol in place. External cooling was the preferred method used by 28 (93.3%) hospitals. The target temperature varied from 32 to 35°C with two (6.7%) ITUs targeting a temperature of 32°C, 11 (36.7%) of 33°C, six (20%) of 34°C, one (10%) of 32 to 33°C, seven (23.4%) of 32 to 34°C and one (10%) of 34 to 35°C. The time of cooling varied between 12 and 48 hours. The cooling period was measured from initiation of cooling by 22 (73.3%) ITUs and from achievement of target temperature by six (20%) ITUs. Two responders were not sure how it was measured. Twenty-five (83.3%) units measured core temperature during the cooling. Passive rewarming was used by 20 (66.6%) responders. Twenty-four (80%) units maintain normothermia post therapeutic hypothermia. From additional aspects of the management of the induced hypothermia, 20 (66.6%) ITUs adjusted drug doses while starting TH, 15 (50%) monitored the depth of sedation, and 17 (56.6%) regularly checked train of four in paralyzed patients. Shivering was treated with sedation and paralysis by 25 (83.3%) of responders. Pregnancy status of all women younger than 50 years old was checked by 10 (33.3%) units. Fifteen (50%) units do not audit the practice regularly.

## Conclusion

There are significant variations in practice between London hospitals which probably reflect the ongoing debate on the optimal management of patients treated with TH. Of note is that 50% of surveyed hospitals do not audit the current practice regularly which may have an impact on the quality and effectiveness of therapeutic cooling.
